# Increased ERp57 Expression in HBV-Related Hepatocellular Carcinoma: Possible Correlation and Prognosis

**DOI:** 10.1155/2017/1252647

**Published:** 2017-03-08

**Authors:** Miao Liu, Lingyao Du, Zhiliang He, Libo Yan, Ying Shi, Jin Shang, Hong Tang

**Affiliations:** ^1^Center of Infectious Diseases, West China Hospital, Sichuan University, Chengdu 610041, China; ^2^Division of Infectious Diseases, State Key Laboratory of Biotherapy and Cancer Center, West China Hospital, Sichuan University and Collaborative Innovation Center for Biotherapy, Chengdu 610041, China

## Abstract

*Aim.* ERp57 is involved in virus induced endoplasmic reticulum stress (ERS) and plays an important role in tumorigenesis. This study aimed to find whether HBV infection altered ERp57 expression and whether ERp57 regulation was involved in hepatitis B virus-related hepatocellular carcinoma (HBV-HCC) genesis.* Materials and Methods.* HBV-HCC tissues, chronic hepatitis B (CHB) liver tissues, and normal liver tissues were acquired. ERp57 expressions in these tissues were detected through immunohistochemistry (IHC). And ERp57 expression in liver cell line L02, HBV replicative liver cell line L02-pHBV4.1, and HCC cell lines were detected through western blot for verification. Then medical data on patients providing HCC tissues were collected and analyzed along with ERp57 expression.* Results.* Higher ERp57 expression was found in HCC and CHB tissues (*p* < 0.001). And HCC cell lines and L02-pHBV4.1 presented higher ERp57 expression as well. In patients, ERp57 expression showed significant differences between death and survival groups (*p* = 0.037). And cumulative survival in patients with higher ERp57 (score *⩾* 8.75) is significantly lower (*p* = 0.009).* Conclusion.* Our study found increased expression of ERp57 in HBV-HCC. Such altered expression could be related to HBV infection and high ERp57 expression may lead to poor prognosis of HBV-HCC patients.

## 1. Background

Hepatitis B virus (HBV) infection is one of the leading causes of hepatocellular carcinoma [1]. Although its mechanisms has been studied for decades, there is still a lot of information that remains unknown. Recent studies have confirmed that some host factors interacting with HBV were involved in viral tumorigenesis, resulting in alternation of host cell biological characteristics [2–4]. The endoplasmic reticulum (ER), where viral DNA replicates and viral proteins are synthesized, could be easily influenced by virus. When virus infects cells, plenty of unfolding or misfolding proteins aggregates in ER to generate a stress. Series of procedures, called endoplasmic reticulum stress (ERS) response, would be triggered to ease it afterwards. And overresponse of ERS would trigger overtranscription of target genes downstream including oncogenes. Endoplasmic reticulum proteins (ERps) play critical roles in ERS and many proteins such as ERp29, ERp72, and calreticulin identified to be ERS concerning [5–8].

In 1999, Oliver found that ERp57 could interact with calreticulin, influencing the folding of newly synthesized proteins [9]. As an important protein disulfide isomerase (PDI), ERp57/GRP58 has been named after abbreviation of endoplasmic reticulum resident protein 57 or 58 kDa glucose-regulated protein. It catalyzes formation, decomposition, and isomerization of disulfide bond, working as a multifunctional protein in kinds of biological procedures [10]. In tumorigenesis, ERp57 presents contradictory roles among different tumors. Low expression of ERp57 in gastric cancer patients would lead to poor prognosis [11]. However, high expression in ovarian cancer patients would result in drug resistance and lead to poor prognosis as well [12]. In liver diseases, ERp57 is suggested to be involved in several hepatic disorders. However, there is no specific study focusing on its roles in HBV-related hepatocarcinogenesis.

So we conducted this study, trying to clarify whether HBV infection altered ERp57 expression and whether ERp57 regulation was involved in hepatitis B virus-related hepatocellular carcinoma (HBV-HCC) genesis.

## 2. Methods

### 2.1. Study Subjects

Tissue sections of HBV-HCC were obtained from pathologic specimen bank of West China Hospital, Sichuan University. Each set of tissues contained a cancer tissue section, an adjacent one and a distal one. Patients providing these samples were part of pathologically diagnosed HBV-HCC patients in West China Hospital in 2012. Their medical data were collected via electronic medical system. Their prognosis was acquired via telephone follow-up.

The chronic hepatitis B (CHB) liver sections were acquired from CHB patients consulting in West China Hospital when liver biopsy was needed to make therapeutic decision. Normal liver sections were acquired from the specimen bank in Department of Forensic Pathology, West China School of Basic and Forensic Medicine, Sichuan University.

HCC cell lines including Huh7, HepG2, and HepG2.2.15, HBV replicative normal liver cell line L02-pHBV4.1, and normal liver cell line L02 were stored in Division of Infectious Diseases, State Key Laboratory of Biotherapy and Cancer Center.

### 2.2. Study Method

#### 2.2.1. Detection of ERp57 Expression in Tissue Samples

Immunohistochemistry (IHC) was used to detect ERp57 expression in tissues. The primary antibody was a rabbit polyclonal IgG to ERp57 (sc-28823, Santa Cruz, USA). And the secondary antibody was part of EnVision™ G2 Systems (Dako, Glostrup, Denmark). With 3,3- diaminobenzidine as reagent to horseradish peroxidase (HRP) linked to the secondary antibody, ERp57 were stained. Nucleus was counterstained with hematoxylin then. After mounting, tissue sections were scored according to Axiotis standard. The percentage of positive cells and its staining intensity were evaluated. The detailed scoring criteria were in Table 1.

#### 2.2.2. Detection of ERp57 Transcription and Expression in Cells

Total RNA was extracted from cells with Trizol (ThermoFisher, USA) and reversely transcribed into cDNA. RT-PCR was applied to detect ERp57 transcription with Fast Start Universal SYBR Green Master in light Cycler 96 (Roche). Detailed information of primers used in RT-PCR was shown in Table 2.

Cells were lysed for total proteins. After quantitation, total proteins were separated with electrophoresis in SDS-polyacrylamide gel and transferred to PVDF membrane. With same multifunctional primary antibody as before and HRP-linked goat against rabbit secondary antibody (ZSGB-BIO, Beijing, China), ERp57 were marked and visualized via chemiluminescent substrate (ThermoFisher, USA) and ChemiDoc™ MP imaging system (Bio-Rad, USA). Then band intensity of ERp57 expression was semiquantified in the imaging system.

### 2.3. Statistical Analysis

All data were processed and analyzed in SPSS 18.00. Enumeration data were described with percentage and analyzed with chi-square test. Measurement data were analyzed for normality first. If the data was normal, it would be described with mean and standard deviation (SD), analyzed with *t*-test. Otherwise it would be described with median and interquartile range (IQR), analyzed with *u* test. Relationship between associated factors, ERp57 expression, and prognosis were analyzed with correlation or regression. The prognosis was analyzed with cumulative survival with Mantel-Cox test.

### 2.4. Ethics Approval and Consent to Participate

The biological samples were acquired for medical or forensic purpose other than our study originally. Samples of HCC tissues were acquired according to surgical resection of tumors. And samples from CHB patients were originally used for assessment of antiviral indications. Normal tissues were originally prepared for medicolegal expertise. Informed consent was signed by patients or their relatives when these actions happened and additional editions were acquired at the same time for samples' further usage of investigational purpose. When follow-up phone calls were made to patients or their relatives, oral permissions on their tissue sections and medical data to be applied in this study were acquired as well. All the procedures were approved and supervised by Ethics Committee of West China Hospital, Sichuan University.

## 3. Results

### 3.1. ERp57 Expression in Different Kinds of Liver Tissues

**Figure 1:**
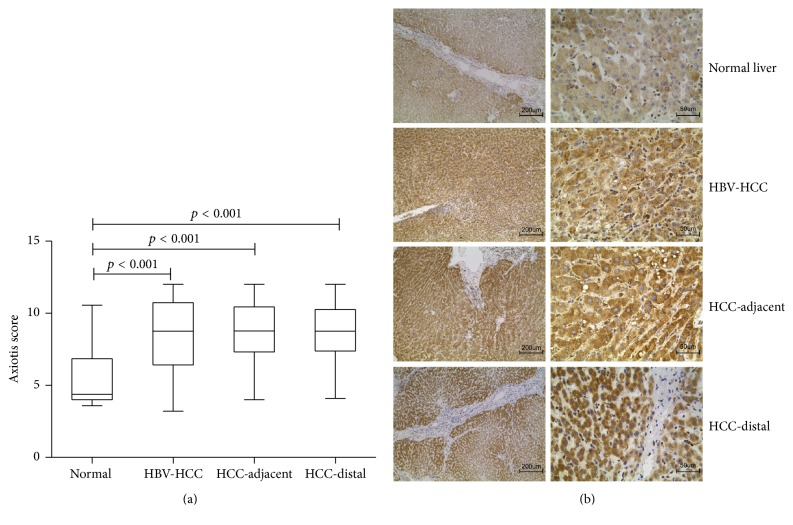
ERp57 expression in HCC liver tissues. (a) Statistical diagram of ERp57 expression score in normal livers and HCC tissues. (b) Representative images of IHC stained ERp57 in normal livers and HCC tissues.

A total of 66 sets of HBV-HCC tissues, 57 CHB tissues, and 16 normal liver tissues were acquired in this study. And positive ERp57 expression was found in all kinds of tissues through IHC. Though no significant difference was found between the median Axiotis score of cancer tissues, adjacent tissues and distal tissues (10.725 ± 2.325 versus 10.45 ± 1.45 versus 10.25 ± 1.36), respectively, their ERp57 expression was significantly higher compared to normal liver tissues (4.375 ± 2.84) (Figure 1). Thus ERp57 expression was altered not only in HBV-HCC tissues but also in HBV-HCC related adjacent and distal tissues. It suggested that the alternation of ERp57 expression could be related to cellular malignant transformation.

**Figure 2:**
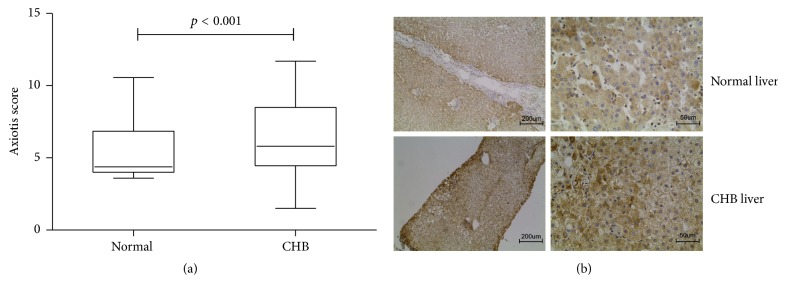
ERp57 expression variation in CHB liver tissues. (a) Statistical diagram of ERp57 expression score in normal livers and CHB liver tissues. (b) Representative images of IHC stained ERp57 in normal livers and CHB liver tissues.

However, the distal tissues showed little manifestation of tumor cells but a status of HBV infection, so HBV infection might be another impact factor to ERp57 expression. To clarify this, CHB liver tissues were compared with normal ones. Its median Axiotis score of ERp57 expression was significantly increased compared to normal liver tissues (4.375 ± 2.84 versus 5.8 ± 4.025, *p* < 0.001) (Figure 2). Such result suggested that HBV infection could be another promoting factor of high ERp57 expression.

### 3.2. ERp57 Expression in Different Kinds of Liver Cell Lines

**Figure 3:**
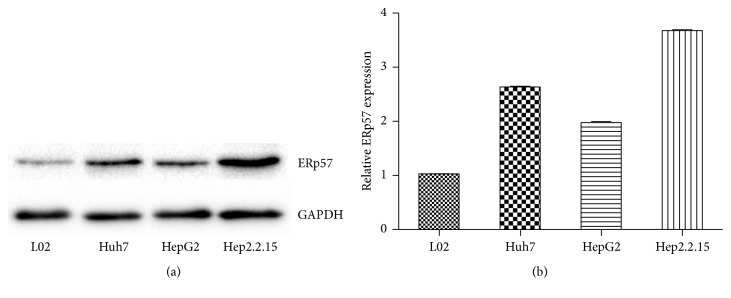
ERp57 expression in normal liver cell line and HCC cell lines. (a) ERp57 expression detected by western blot in different cell lines with GAPDH as internal reference. (b) Statistical diagram of Quantity One captured western blot detected ERp57 expression data. The ERp57/GAPDH ratios of other cell lines were transformed into relative ratio according to the ERp57/GAPDH ratio of L02 cells as “1.”

Experimental results in cell lines confirmed the correlation between cellular malignant transformation, HBV infection, and ERp57 expression. ERp57 expression in HCC cell lines including Huh7, HepG2, and HepG2.2.15 was significantly increased compared to normal liver cell L02, proving that cellular malignant transformation promoted ERp57 expression. As preconceived, the ERp57 expression in Huh7 and HepG2 was only increased moderately. But it was increased strongly in HepG2.2.15 cells which mimicked a status of HBV-HCC (Figure 3).

**Figure 4:**
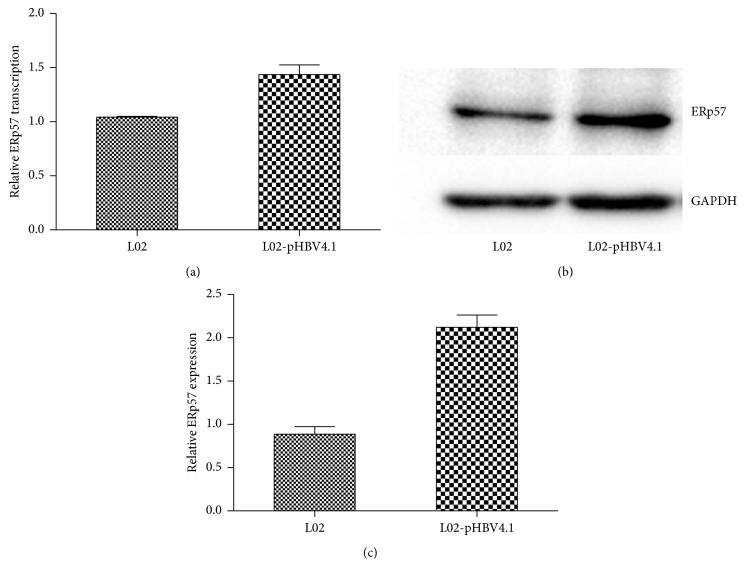
Transcription and expression level of ERp57 in normal liver cell line L02 and HBV replicative liver cell line L02-pHBV4.1. (a) RT-PCR detected relative ERp57 mRNA level in L02 and L02-pHBV4.1 with GAPDH as internal reference. (b) ERp57 expression detected by western blot in L02 and L02-pHBV4.1 with GAPDH as internal reference. (c) Statistical diagram of Quantity One captured western blot detected ERp57 expression data. The ERp57/GAPDH ratios of HBV infected cell line, L02-pHBV4.1, was transformed into relative ratio according to the ERp57/GAPDH ratio of L02 cells as “1.”

L02-pHBV4.1 was established by stably transfecting HBV replicative plasmid pHBV4.1 into L02. It mimicked the status of HBV infected liver without inflammatory cell infiltration and it is applicable to investigate the influence from HBV to host cytokines. In this cell, ERp57 transcription and expression were significantly increased compared to L02, suggesting that HBV did promote ERp57 expression and such influence could be relevant to ERp57 transcription (Figure 4).

### 3.3. Correlation between ERp57 Expression in HBV-HCC Tissues and Prognosis

In the 66 HBV-HCC patients providing tissues, male patients took the minority (*N* = 8). The mean age was 50.23 ± 11.60 years old. Alpha fetal protein (AFP) in these patients was distributed widely (0.92-495383.00 ng/ml). Most patients only showed moderately elevated ALT (47 ± 38.5 IU/L). Most patients (*N* = 52) were diagnosed as HBeAg negative CHB. Viral loads of the whole crowed located at a relatively low level (500 ± 6167.5 IU/mL). According to radiological examinations, surgery records, and pathological reports, Ishake score of the 66 distal tissues showed a median value of 5 ± 2, implying most patients had background of cirrhosis. And 46 patients suffered tumors with size *⩾* 5 cm. Moreover, high differentiated tumor was found in 6 patients, while moderate differentiation was found in 50 patients and poor differentiation was found in 10 patients. Capsule invasion, margin involvement, tumor thrombus, and metastasis were found in 39, 3, 21, and 7 patients, respectively. Till the date of follow-up call made (December, 2015), 28 patients survived and 19 patients died of HCC related complications. The other 19 patients were lost to follow-up (Table 3).

Patients' biological characteristics and factors related to the disease status were analyzed for their correlation to ERp57 expression in cancer tissues. Through univariate analysis, occurrence of tumor thrombus and prognosis were identified as two significant factors related to ERp57 expression in cancer tissues in this crowd (*p* = 0.021 and 0.037) (Table 3). As patients with tumor thrombus would encounter poor prognosis more frequently, the significant relationship between ERp57 expression and tumor thrombus also reminded that ERp57 was an essential factor related to prognosis.

**Figure 5:**
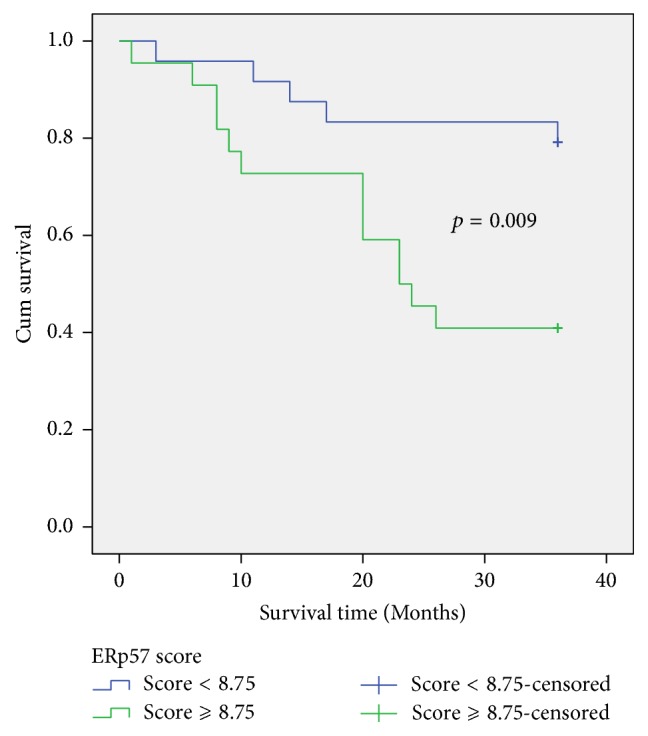
Cumulative Survival in two groups divided by ERp57 expression. Two groups were divided by cut-off value (Axiotis score = 8.75). Blue curve represented the group with ERp57 score < 8.75, while the green curve represented the group with ERp57 score *⩾* 8.75. According to Mantel-Cox test, cumulative survival of these two groups showed significant difference (*p* = 0.009).

So we excluded patients lost to follow-up, dividing the rest of them into two groups according to their prognosis (death or survival). The occurrence of tumor thrombus was obviously more frequent in patients with poor prognosis (*p* = 0.003) and ERp57 expression did show significant differences between the death and survival groups (10.6 + 4.15 versus 8.025 + 3.4, *p* = 0.037) (Table 4). Then we calculated an ROC curve to identified a cut-off value (Axiotis score = 8.75) and divided whole crowd into two groups according to the criterion. In the follow-up period of 36 months, the cumulative survival in group with score *⩾* 8.75 is significantly lower compared to group with score < 8.75 (*p* = 0.009) (Figure 5). All these results suggested that HBV-HCC patients with higher expression of ERp57 in cancer tissues would encounter shorter life expectancy.

## 4. Discussion

The solid tumor cells live in an acid environment with low oxygen because tumor itself usually develops faster than its vascularization [5]. In such an environment, ERS is essential to tumor cells. It activates series of signaling transduction pathway to assist the cell adaptive survival. For example, ERp57 in cells works coordinately with calreticulin to influence folding of newly synthesized proteins. Upregulated ERp57 would induce unfolded protein response (UPR), one type of ERS, to prevent export of unfolded proteins. Through such way, tumor cells could maintain their cellular homeostasis for survival [6, 7]. And when ERp57 joins in transcriptional complex assembly along with STAT3, it could be involved in nuclear signaling pathway to promote cell proliferation. However, ERp57 also participates in formation of major histocompatibility complex class I (MHC I) to influence the immunogenicity of tumor cells. So upregulated ERp57 would accelerate maturation of dendritic cells, enhancing host surveillance and reducing production of negative regulators to antitumor immunity [13].

The controversial function in the mechanism endows ERp57 complicated roles in different kinds of tumors [11, 12, 14]. However, the exact role it played in HBV-HCC remained to be learned. In our study, we found that no matter in HBV-HCC cancer tissues, adjacent tissues, or distal tissues, higher expression of ERp57 was found compared to normal liver tissue. Interestingly, ERp57 expression among the three kinds of HBV-HCC related tissues showed no differences. Such result implied that ERp57 was increased in cell transition to malignancy. And its expression might have been upregulated already before the cells were fully transformed. The fact that cell lines derived from HCC presented higher ERp57 expression than normal liver cells also verified the correlation between malignant transition and ERp57 expression.

The background of HBV infection no matter in HBV-HCC tissues, especially the distal ones, or in HepG2.2.15 cells suggested a possible correlation between HBV infection and ERp57 expression. HBV has been confirmed as a tumorigenesis virus for decades [15]. As the endoplasmic reticulum is an important site of virus replication and progeny virus assembly, plenty of viral proteins aggregate in the ER when virus infected ER, triggering ERS to upregulated ER proteins expression [16]. Previous study has found HCV could upregulate ER proteins such as GRp78 and GRp94 in the liver [17]. As another hepatotropic virus, HBV was confirmed to be involved in the regulation of ER proteins in our study. ERp57 expression was increased both in liver tissues and in cell lines if there was HBV infection.

It was interesting that when we analyzed the relationship between serum HBV viral load and the ERp57 expression in HCC patients, no significant result was found (*p* = 0.480). Such phenomenon seemed not to make sense as HBV was considered to be one of the ERp57 inducers. However, the extremely low level of viremia in HCC patients would be an explanation. Viruses need proper intracellular environment to accomplish their life cycles. As a result, the alternation in HCC cells as well as the host immunity of HCC patients would inhibit the viral replication [18]. In our study, the median viral load is 2log⁡10 IU/mL. Statistically, extremely low serum HBV DNA would decrease the difference of serum viral load among patients and lead to an insufficient analysis. Moreover, the cells had been malignantly transformed already. As we found that HCC cell lines presented higher expression of ERp57 (Figure 3), the impact from HBV to ERp57 expression would be covered by the malignant transformation to some degree. In recent studies, scientists proved again that the serum HBV DNA and intracellular cccDNA and total DNA were significantly higher in CHB patients than in HCC patients, and they also found an inconsistency between serum viral DNA and intracellular viral DNA in HCC patients [19]. So significant relationship might be found if intracellular HBV DNA could be detected other than serum HBV DNA.

ERp57 expression was reported to be associated with prognosis of patients. In gastric cancer where ERp57 presented as a tumor inhibitor, low expression of ERp57 results in poor prognosis [11]. However, the situation was quite different in our study. When possible factors related to ERp57 expression were analyzed, the existence of tumor thrombus and prognosis were the only two significant ones. Portal vein tumor thrombus in HCC is a proved poor prognostic factor [20]. Patients with portal vein tumor thrombus were reported to encounter a median survival time about 4 months [21, 22]. So the result suggested a correlation between high ERp57 expression and poor prognosis. Moreover, when patients were grouped according to their prognosis, ERp57 was increased significantly in the death group. And the cumulative survival analysis revealed that patients with higher ERp57 expression would have shorter survival time. These results proved that ERp57 was involved in the prognosis of HBV-HCC patients and its influence on the occurrence of tumor thrombus could be one way. Other studies supported our findings. A newly reported research demonstrated that* Antrodia cinnamomea* (EEAC), a Chinese herb, decreased ERp57 to suppress HCC migration. Downregulation of ERp57 by siRNA effectively inhibit transwell immigration of HCC cells. It suggested that ERp57 could affect cell proliferation and migration [23].

ERp57 locates in cytoplasm, nucleus, and endoplasmic reticulum. Its dynamic subcellular localization is considered as a key factor to explain its complex action in tumorigenesis. Oncogene activation is vastly related to ERp57 immigration into nucleus as part of transcription complex. As part of STAT-3 transcription complex, ERp57 translocated into nucleus to modulate intracellular gene expression mediated by TORC1 and TORC2, interfering Ref-A1 related DNA repair [24, 25]. And ERp57 also combines with nuclear factor *κ*B (NF-*κ*B), altering its subcellular location [13]. So the correlation between dynamic subcellular localization of ERp57 and its multiple biological function is worth further investigation.

## 5. Conclusion

Our study found increased expression of ERp57 in HBV-HCC. Such altered expression could be related to HBV infection and high ERp57 expression may lead to poor prognosis of HBV-HCC patients. It provides information on the role ERp57 played in HBV-HCC genesis, guiding us a direction to further investigations in its mechanism.

## Figures and Tables

**Table 1:** Detailed criteria of Axiotis Score^*∗*^.

Percentage score		Intensity score	
0	0~10% positive cells	0	No color
1	11~25% positive cells	1	Yellow
2	26~50% positive cells	2	Brown
3	51~75% positive cells	3	Tan
4	76~100% positive cells		

^*∗*^The sum of the two scores equaled the sum score. And five different sum scores from random view under 400x magnification were acquired for a mean sum score. The assessments were implemented by two pathologists unaware of the tissue section arrangement. If there were differences in their opinions, extra mean would be calculated with the two mean sums for final score.

**Table 2:** Sequences of primers used in RT-PCR.

Primer name	Sequence
ERp57 forward primer	5′-CTCCTCGCCTCCGCCTCAGA-3′
ERP57 reverse primer	5′-AGCCCACCACCGAGGCATCT-3′
GAPDH forward primer	5′-ACCCACTCCTCCACCTTTGA-3′
GAPDH reverse primer	5′-CTGTTGCTGTAGCCAAATTCGT-3′

**Table 3:** Univariate analysis of factors related to ERp57 expression in HCC tissue sections.

Factors	Frequency/media (mean) ± IQR(SD)	Mann–Whitney test (*Z*)/Pearson test (*r*)/Kruskal-Wallis test (*X*^2^)	*P* value
Gender (male/female)	8/58	−0.079	0.937
Age (year)	50.23 ± 11.60	−0.169	0.176
AFP (ng/ml)	198.3 ± 1690.84	0.213	0.091
ALT (IU/L)	47 ± 38.5	−0.105	0.408
HBeAg (positive/negative)	14/52	−1.044	0.297
HBVDNA (IU/mL)	500 ± 6167.5	0.089	0.480
Ishak Score	5 ± 2	−0.210	0.090
Tumor Size (<5 cm/*⩾*5 cm)	20/46	−1.487	0.137
Differentiated degree (poor/moderate/high)	10/50/6	−1.347	0.178
Capsule invasion (positive/negative)	39/27	−1.742	0.081
Margin involvement (positive/negative)	3/63	−0.893	0.372
Tumor thrombus (positive/negative)	21/45	−2.3	0.021
Metastasis (positive/negative )	7/59	−1.063	0.288
Prognosis (death/survival/lost to follow-up)	19/28/19	−2.083	0.037

**Table 4:** Univariate analysis of factors related to prognosis in patients within follow-up (*N* = 47).

Factors	Death (*N* = 19)	Survival (*N* = 28)	Mann–Whitney test (*Z*)/Pearson test (*r*)/Kruskal-Wallis test (*X*^2^)	*P* value
Gender (male/female)	17/2	25/3	−0.000	1.000
Age (year)	46 ± 25	50.5 ± 13.75	−0.651	0.515
AFP (ng/ml)	528.8 ± 3064.72	93.62 ± 1153.70	−0.902	0.367
ALT (IU/L)	37 ± 23.5	46 ± 28	−1.206	0.228
HBeAg (positive/negative)	2/17	8/20	2.201	0.168
HBVDNA (IU/mL)	500 ± 0	500 ± 10775	−1.192	0.233
Ishak Score	4 ± 2	5 ± 2	−0.906	0.365
Tumor Size (<5 cm/*⩾*5 cm)	5/14	11/17	0.848	0.357
Differentiated degree (poor/moderate/high)	3/16/0	3/20/5	3.863	0.145
Capsule invasion (positive/negative)	9/10	15/13	0.174	0.676
Margin involvement (positive/negative)	2/17	1/27	0.916	0.338
Tumor thrombus (positive/negative)	10/9	3/25	9.940	0.003
Metastasis (positive/negative)	3/16	1/27	2.170	0.289
Axiotis score of ERp57 expression in tissue sections	10.6 ± 4.15	8.025 ± 3.4	−2.083	0.037
